# EEG neurofeedback treatments in children with ADHD: an updated meta-analysis of randomized controlled trials

**DOI:** 10.3389/fnhum.2014.00906

**Published:** 2014-11-13

**Authors:** Jean-Arthur Micoulaud-Franchi, Pierre Alexis Geoffroy, Guillaume Fond, Régis Lopez, Stéphanie Bioulac, Pierre Philip

**Affiliations:** ^1^Solaris, Unité de Neurophysiologie, Pôle de Psychiatrie Universitaire, Hôpital Sainte-MargueriteMarseille, France; ^2^Institut de Neurosciences Cognitives de la Méditerranée, INCM-CNRS UMR 6193Marseille, France; ^3^Inserm, UMR-S 1144Paris, France; ^4^AP-HP, GH Saint-Louis - Lariboisière - Fernand Widal, Pôle NeurosciencesParis, France; ^5^UMR-S 1144, Université Paris Descartes and Université Paris DiderotParis, France; ^6^Fondation FondaMentalCréteil, France; ^7^Université Paris Est-Créteil, Pôle de Psychiatrie du Groupe des Hôpitaux Universitaires de Mondor, DHU Pe-psy, INSERM U955, Eq Psychiatrie Génétique, Réseau des Centres Experts Schizophrénie de FranceCréteil, France; ^8^Centre de Référence National Narcolepsie-hypersomnie Idiopathique, Unité des Troubles du Sommeil, CHU Gui de ChauliacMontpellier, France; ^9^NSERM U1061Montpellier, France; ^10^Centre Hospitalier Charles Perrens, Pôle Universitaire de Psychiatrie de l’Enfant et de l’AdolescentBordeaux, France; ^11^USR CNRS 3413 SANPSY, Clinique du Sommeil, CHU Pellegrin, Université de BordeauxBordeaux, France

**Keywords:** attention deficit hyperactivity disorder, neurofeedback, randomized controlled trial, learning, practice guidelines

## Abstract

**Objective**: We undertook a meta-analysis of published Randomized Controlled Trials (RCT) with semi-active control and sham-NF groups to determine whether Electroencephalogram-neurofeedback (EEG-NF) significantly improves the overall symptoms, inattention and hyperactivity/impulsivity dimensions for probably unblinded assessment (parent assessment) and probably blinded assessment (teacher assessment) in children with Attention Deficit Hyperactivity Disorder (ADHD).

**Data sources**: A systematic review identified independent studies that were eligible for inclusion in a random effects meta-analysis.

**Data extraction**: Effect sizes for ADHD symptoms were expressed as standardized mean differences (SMD) with 95% confidence intervals.

**Results**: Five identified studies met eligibility criteria, 263 patients with ADHD were included, 146 patients were trained with EEG-NF. On parent assessment (probably unblinded assessment), the overall ADHD score (SMD = −0.49 [−0.74, −0.24]), the inattention score (SMD = −0.46 [−0.76, −0.15]) and the hyperactivity/impulsivity score (SMD = −0.34 [−0.59, −0.09]) were significantly improved in patients receiving EEG-NF compared to controls. On teacher assessment (probably blinded assessment), only the inattention score was significantly improved in patients receiving EEG-NF compared to controls (SMD = −0.30 [−0.58, −0.03]).

**Conclusions**: This meta-analysis of EEG-NF in children with ADHD highlights improvement in the inattention dimension of ADHD symptoms. Future investigations should pay greater attention to adequately blinded studies and EEG-NF protocols that carefully control the implementation and embedding of training.

## Introduction

The techniques of neurofeedback (NF) enable a patient to train him or herself to self-regulate a single measure of brain activity (Coben and Evans, 2011; Micoulaud-Franchi et al., 2014). Brain activity can be measured through electroencephalography (EEG); the technique is thus called EEG-NF. EEG-NF training aims to achieve self-control over specific aspects of electrical brain activity through real-time feedback and positive reinforcement and implement these self-regulation skills in daily life (Heinrich et al., 2007; Gevensleben et al., 2012). There is growing interest in the use of neurofeedback treatment in Attention Deficit Hyperactivity Disorder (ADHD) by providing strategies for better self-regulation and management of some disturbances of the disorder (Gevensleben et al., 2012; Arns et al., 2014; Vollebregt et al., 2014b). Nevertheless, NF effectiveness is one of the most debated subjects in this area at the moment (Gevensleben et al., 2012; Arns and Strehl, 2013; Sonuga-Barke et al., 2013b; Arns et al., 2014; Cannon et al., 2014; Dagenais et al., 2014; van Dongen-Boomsma, 2014; van Dongen-Boomsma et al., 2014).

Despite the significant effects of probably not blinded assessment (i.e., an assessment made by an individual likely to be not blind to treatment, which was in most cases the parent assessment) (Arns et al., 2009), a recent meta-analysis by Sonuga-Barke et al. (2013a) reported a trend of only four Randomized Controlled Trials (RCT) with semi-active control (i.e., cognitive remediation or electromyographic (EMG)-biofeedback) and sham-NF groups (i.e., control conditions where everything is identical to the EEG-NF, except that in this case the feedback is not related to brain activity) (Arns et al., 2014), with “probably blinded assessment” (i.e., assessment made by an individual likely to be blind to treatment, which was in most cases assessment made by a teacher) (Gevensleben et al., 2009b; Bakhshayesh et al., 2011; Lansbergen et al., 2011; Steiner et al., 2011). Moreover, the effect of the total score on scale evaluating overall ADHD symptoms with probably blinded assessment was small (SMD = −0.29 [−0.61, 0.02], *p* = 0.07) (Sonuga-Barke et al., 2013a). This result was in line with the previous meta-analysis by Arns et al. (2009) that observed smaller effects in better-controlled studies (Arns et al., 2009).

Since this later meta-analysis, further RCTs were published (Arns et al., 2014); because of the methodological issues regarding blinded or unblinded assessment (by parents or teachers) (Arnold et al., 2013), we decided to further examine the efficacy of EEG-NF on ADHD in an updated meta-analysis. In addition, the meta-analysis by Sonuga-Barke et al. (2013a) did not analyze the inattention and hyperactivity/impulsivity dimensions separately, which define the three primary subtypes of ADHD: the predominately inattentive type, the predominantly hyperactive/impulsive type and the combined type (American Psychiatric Association, 2000; Polanczyk et al., 2007). Thus, we perform the present meta-analysis on overall ADHD symptoms as well as the inattention and hyperactivity/impulsivity dimensions for both probably unblinded assessment (parent assessment) and probably blinded assessment (teacher assessment). Thus, the aim of this study was to focus on recent major developments in the field of NF and ADHD in order to complete and update the meta-analysis of Sonuga-Barke et al. (2013a) by including further RCTs, published after this later meta-analysis with semi-active control and sham-NF groups to compare the NF intervention with an intervention that controls for the non-specific effects of EEG-NF (Arnold et al., 2013; Arns et al., 2014).

## Methods

We followed the Preferred Reporting Items for Systematic Reviews and Meta-analyses (PRISMA) recommendations to undertake the search and analysis of the international scientific literature (Moher et al., 2009).

We searched PubMed, Embase and Google Scholar databases for publications between April 2012, the date of search finalization of the previous meta-analysis (Sonuga-Barke et al., 2013a) and August 2014. The following MESH terms were used: (“Neurofeedback” OR “EEG Biofeedback”) AND (“ADHD” OR “attention-deficit/hyperactivity disorder”). We also examined the citation lists of identified publications for additional studies, used the related articles function of the PubMed database. English language publications reporting a RCT were eligible for inclusion. Studies were included if they met the following criteria:

1. Design: randomized controlled trials (RCT).
2. Intervention: standard protocol EEG-NF with Theta/Beta Ratio training—TBR (or likely to standard TBR training) or Slow Cortical Potentials (SCP) training.
3. Control group: semi-active (i.e., cognitive remediation and EMG-biofeedback) and sham-NF.
4. Participants: participants with an established clinical diagnosis of ADHD thanks to DSM or CIM criteria.
5. Evaluation of ADHD severity based on a validated scale with probably blinded assessment (teacher assessment) data available.
6. No secondary analyses of previously included trials.

Data was independently extracted into a standard electronic form by two authors (Jean-Arthur Micoulaud-Franchi and Pierre A. Geoffroy): first author name, date of publication, country, EEG-NF protocol, number of session, duration of session, electrode positions, manual or automatic threshold reward, session of transfer learning strategies in daily life, control protocols, sample size, mean age, percentage of ADHD males included, percentage of co-administration of methylphenidate, parent and teacher ADHD assessment (overall, inattention and hyperactivity/impulsivity scores).

We calculated a standardized mean difference (SMD) with 95% confidence intervals (CIs) for each study, defined as the difference in pre-post treatment mean changes between the two groups (ADHD with EEG-NF vs. control groups) divided by the pooled standard deviation of the measurements, as previously performed by Sonuga-Barke et al. (2013a). Random effects modeling for pooled effect sizes (ES) were used because it provides a more conservative ES estimate (Hedges and Olkin, 1985; DerSimonian and Laird, 1986). The SMDs were interpreted in a similar manner to Cohen’s d (0.2 = small ES; 0.5 = medium ES; 0.8 = large ES). Confidence limit ratios (CLR = upper-to-lower confidence limit ratio) were calculated for significant CIs in order to estimate the precision and the random error (Poole, 2001). The I^2^ statistic was used to quantify heterogeneity, with the values of 25%, 50% and 75% reflecting a small, medium or high degree of heterogeneity, respectively (Higgins et al., 2003). We used funnel plots to estimate by visual inspection the risk of bias (Borenstein et al., 2009). Forest plots were generated to show SMD with corresponding CIs for each study and the overall estimate of pooled random effects. We conducted two subgroups analyses to determine the impact of probably blinded assessment (teacher assessment) on ES estimates for EEG-NF effectiveness. Because sensitivity analysis to test for EEG-NF and clinical characteristics effects was not possible because of the small number of trials, we tested the correlation between ES and mean age, percentage of male, percentage of patient treated with methylphenidate with Spearman rank correlations. All analyses were performed with Review Manager 5.2 software (Cochrane Collaboration, Copenhagen, Denmark) and SPSS software (Version 18, PASW Statistics).

## Results

### Results of the literature search

Twelve RCTs were published since April 2012. We excluded one study with a non-standard EEG-NF protocol (Arnold et al., 2013), five studies with non-semi-active or sham-NF control groups (i.e., treatment as usual or methylphenidate) (Li et al., 2013; Ogrim and Hestad, 2013; Bink et al., 2014; Meisel et al., 2014) and one study with no available probably blinded assessment data (Duric et al., 2012). Two studies were excluded because there were secondary analyses of already included RCTs (Steiner et al., 2014a; Vollebregt et al., 2014a).

Three studies from April 2012 to August 2014 (van Dongen-Boomsma et al., 2013; Maurizio et al., 2014; Steiner et al., 2014b) were eligible for inclusion. The previous meta-analysis by Sonuga-Barke et al. (2013a) included four RCTs (Gevensleben et al., 2009b; Bakhshayesh et al., 2011; Lansbergen et al., 2011; Steiner et al., 2011). We excluded studies that would lead us to pool data to avoid including the same patients more than once. Indeed, two studies eligible for inclusion in the present meta-analysis (van Dongen-Boomsma et al., 2013; Steiner et al., 2014b) were continuations of pilot studies included in the meta-analysis of Sonuga-Barke et al. (2013a) (Lansbergen et al., 2011; Steiner et al., 2011). These two pilot studies were not included in the present meta-analysis.

At the end of this RCT selection process, five studies were retained for quantitative analysis: two from the previous meta-analysis of Sonuga-Barke et al. (2013a) (Gevensleben et al., 2009b; Bakhshayesh et al., 2011) and three recently published RCTs (van Dongen-Boomsma et al., 2013; Maurizio et al., 2014; Steiner et al., 2014b).

### Results of the meta-analysis

#### Studies and populations characteristics

Overall, 263 patients with ADHD were included vs. 179 in the meta-analysis of Sonuga-Barke et al. (2013a), the mean age range was 8.4–10.6 years, the range of the male percentages was 67.6–96.3% and the range of the children percentages taking methylphenidate was 0–50%. One hundred and forty-six patients vs. 103 in the meta-analysis of Sonuga-Barke et al. (2013a) were trained with EEG-NF. Four trials studied TBR training (Bakhshayesh et al., 2011; Maurizio et al., 2014; Steiner et al., 2014b) or likely to standard TBR training (van Dongen-Boomsma et al., 2013), one used the combination of both: TBR training and training of SCP (Gevensleben et al., 2009b). Sixty-nine controls received cognitive remediation (Gevensleben et al., 2009b; Steiner et al., 2014b) and 48 controls received sham-NF (van Dongen-Boomsma et al., 2013) or EMG biofeedback (Bakhshayesh et al., 2011; Maurizio et al., 2014). Three differents ADHD scales were used: the German ADHD Rating Scale, the ADHD Rating Scale and the Conners’ Rating Scale. Table 1 summarizes the characteristics of the included studies.

**Table 1:** **Summary of characteristics of studies included in the meta-analysis of randomized controlled trials of EEG-NF treatments in ADHD**.

		EEG-NF characteristics							*N*		Clinical characteristics		
	Country	Number of sessions	Duration of sessions	Electrode positions	Protocol	Rewards	Transfer learning strategies in daily life	Control	EEG-NF	Control	Age (years; mean, SD, [range])	Male %	Methyphenidate %	ADHD measures
Gevensleben et al. (2009b)	DE	36	50	Cz	TBR and SCP	Manual	Yes	Cognitive remediation	59	35	9.1 (1.3) [8–12]	89.1	0	FBB-HKS
Bakhshayesh et al. (2011)	DE	30	30	FCz-CPz	TBR	Manual	No	EMG biofeedback	18	17	9.6 (2.2) [6–14]	72	22	FBB-HKS
van Dongen-Boomsma et al. (2013)	NL	30	45	F3-F4 Fz C3-C4 or P3-P4	Likely to standard TBR protocol	Manual	Yes	Sham neurofeedback	22	19	10.5 (2.2) [8–15]	86.4	50	ADHD-RS
Steiner et al. (2014b)	USA	40	45	Close to Cz	TBR	Automatic	No	Cognitive remediation	34	34	8.4 (1.1) [Not available*]	67.6	44.4	Conners-RS
Maurizio et al. (2014)	SW	36	12–32	Tomographic EEG on ACC	TBR	Manual	Yes	EMG biofeedback	13	12	10.6 (1.3) [8.5–12.9]	96.3	7.6	FBB-HKS

EEG-NF = EEG Neurofeedback; TBR= Theta/Beta Ratio; SCP = Slow Cortical Potential; ACC = Anterior Cingulate Cortex; FBB-HKS = German ADHD Rating Scale (Parent and Teacher); ADHD-RS = ADHD Rating Scale (Parent and Teacher); Conners-RS = Conners’ Rating Scale (Parent and Teacher). *Second and fourth grade students in public elementary schools.

#### Effects of EEG-NF on parent assessment (probably no-blinded assessment)

**Figure 1:**
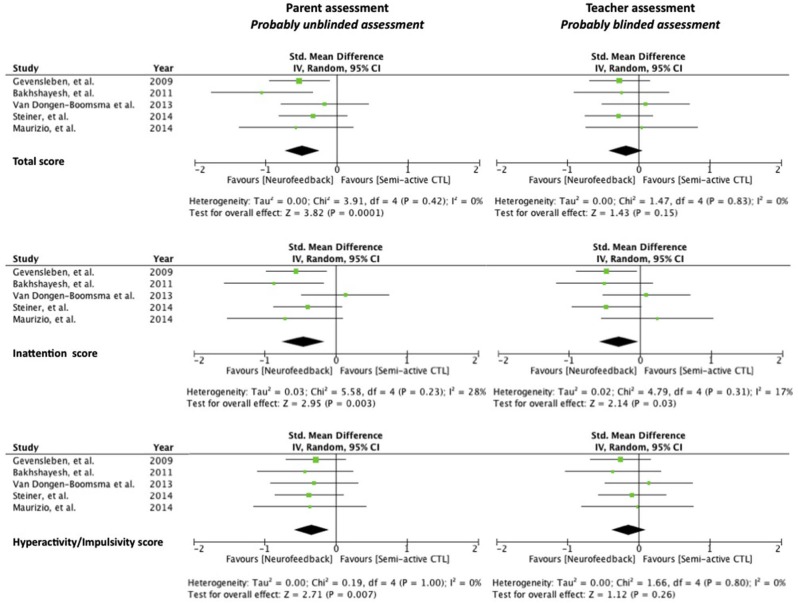
**Forest plots with Standardized Mean Difference (SMD), effect size, and homogeneity statistics for the meta-analysis examining total scores of ADHD symptoms, inattention dimension and hyperactivity/Impulsivity dimension assessed by parent (left) and by teacher (right)**.

The overall ADHD score (SMD = −0.49 [−0.74, −0.24], CLR = 3.08, *p* < 0.001), the inattention score (SMD = −0.46 [−0.76, −0.15], CLR = 3.04, *p* = 0.003) and the hyperactivity/impulsivity score (SMD = −0.34 [−0.59, −0.09], CLR = 6.55, *p* = 0.007) were significantly improved in patients receiving EEG-NF compared to controls. The three associated funnel plots were reasonably symmetrical excluding publication biases (Figure 1).

#### Effect of EEG-NF on teacher assessment (probably blinded assessment)

The inattention score was significantly improved in patients receiving EEG-NF compared to controls (SMD = −0.30 [−0.58, −0.03], CLR = 19.33, *p* = 0.03). No significant effect was found on the overall ADHD score (SMD = −0.18 [−0.42, 0.07], *p* = 0.15) and the hyperactivity/impulsivity score (SMD = −0.14 [−0.39, 0.10], *p* = 0.26). The three associated funnel plots were reasonably symmetrical excluding publication biases (Figure 1).

#### Sensitivity analysis to test for medication effects

A significant correlation was found between the ES on the overall ADHD score assessed by teacher and percentage of patient treated with methylphenidate (rs[5] = 0.9, *p* = 0.037). The more the effect size is negative (i.e., in favor of EEG-NF), the less the percentage of patient treated with methylphenidate. No other significant correlation between ES and EEG-NF and clinical characteristics was found.

## Discussion

The major findings of this updated meta-analysis are that: (i) EEG-NF significantly improves the ADHD total score on a parent-assessment scale with a medium effect size of −0.49; (ii) EEG-NF significantly improves both the inattention and hyperactivity/impulsivity dimensions on a parent-assessment scale with medium effect sizes of −0.46 and −0.34, respectively; and (iii) EEG-NF significantly improves the inattention dimension on a teacher-assessment scale with a smaller effect size of −0.30.

Our results confirmed the findings provided by the meta-analysis of Sonuga-Barke et al. (2013a) on the overall ADHD score with a medium effect size of −0.59 on a probably blinded assessment and of −0.29 on a probably unblinded assessment. Note that for overall scores on a probably unblinded assessment, the CLR was 3.08 similar as in Sonuga-Barke et al. (2013a). In our study, CLR was 3.04 for the inattention dimension with a probably unblinded assessment, and was higher with a probably blinded assessment (19.33). This result indicates that probably blinded assessment is influenced more by random error and is more unstable than unblinded assessment. Thus, the evidence supporting EEG-NF interventions was influenced by the probable blindness status of the assessor (probably unblinded parent vs. probably blinded teacher). These results suggest that EEG-NF should be evaluated by at least one probably blinded assessor.

The methodological strength and novelty of the present updated meta-analysis was to combine stringent inclusion criteria similar to the meta-analysis of Sonuga-Barke et al. (2013a) with the additional consideration of the inattention and hyperactivity/impulsivity dimensions. These precautions allow us to observe the effects of evidence-supporting EEG-NF on inattention symptoms in ADHD in both probably unblinded parents and probably blinded teacher assessments with similar effect sizes. On the contrary, EEG-NF was found to be effective in hyperactivity/impulsivity only in probably unblinded parent assessments. These results emphasize those of Arns et al. (2009), who observed a smaller size effect for the hyperactivity dimension than for the inattention dimension. Though moderate, the effect size remains significant in our meta-analysis compared to the large effect size observed by Arns et al. (2009). It could be explained by the fact that some trials included in our meta-analysis attempt to blind parents to treatment allocation by using sham NF (van Dongen-Boomsma et al., 2013) or EMG biofeedback with comparable electrode placement to EEG-NF (Bakhshayesh et al., 2011; Maurizio et al., 2014). This improved blinded methodology can diminish the risk of rater bias concerning the placebo effect of electronic devices (Schwitzgebel and Traugott, 1968; Stroebel and Glueck, 1973) and should thus be encouraged in further studies (Arnold et al., 2013; Arns et al., 2014).

The effect size in favor of EEG-NF to treat the inattention dimension of ADHD confirms the standard target of the EEG-NF protocol. EEG-NF, through the TBR or SCP training provides immediate feedback on how the brain is focusing. Thus, these protocols are classically known to reinforce the state of attention (focused and attentive but relaxed) (Monastra et al., 2005; Sherlin et al., 2011; Arns et al., 2014). The significant correlation between the teacher-assessed overall ADHD score and methylphenidate treatment could also be explained by the fact that methylphenidate decreases the TBR in children, exhibiting a positive medication response (Loo et al., 1999). As it was determined that low TBR at baseline was a negative predictor for EEG-NF (Gevensleben et al., 2009a; Arns et al., 2012), this pharmacological EEG enhancement could reduce the possibility of training on this parameter during a session of EEG-NF (Sherlin et al., 2011). Thus, further studies should analyze the relationship between TBR at baseline and the enhancement of inattention after an EEG-NF intervention and the effect of methylphenidate on performance during EEG-NF training in children with ADHD.

The principal limitations of our meta-analysis include the small number of studies, the relatively small number of subjects enrolled in the individual studies, and the heterogeneous methodology concerning the characteristics of the EEG-NF protocols (Table 1). As we conducted an updated meta-analysis of Sonuga-Barke et al. (2013a) by including further RCTs according to similar criteria of inclusion and exclusion, we included only a small number of studies insufficient in order to explore potential reasons of heterogeneity between other studies with less conservative inclusion criteria. Moreover, the inclusion of the van Dongen-Boomsma et al. (2014) study in our meta-analysis could be discussed in line with the debate concerning the inclusion of the Lansbergen et al. (2011) study in the meta-analysis of Sonuga-Barke et al. (2013a) (Arns and Strehl, 2013; Sonuga-Barke et al., 2013b; Arns et al., 2014; Cannon et al., 2014; van Dongen-Boomsma, 2014; van Dongen-Boomsma et al., 2014). The EEG-NF protocol of the pilot study of van Dongen-Boomsma et al. (2013) was considered to be non-standard (Arns and Strehl, 2013; Arns et al., 2014; Cannon et al., 2014). However, we decided to include the van Dongen-Boomsma et al. (2013) study because two changes were made (manually adjusted reward thresholds and transfer learning strategies in daily life) that bring their EEG-NF protocol closer to a standard TBR protocol (Sherlin et al., 2011).

The inclusion of the Maurizio et al. (2014) study in our meta-analysis could be also a subject of discussion because it uses a tomographic EEG-NF that is rarely used in a clinical context. However, we decided to include this study because this training protocol was very close to standard TBR protocol on scalp-level EEG-NF. The main difference was the higher spatial resolution with tomographic EEG-NF. Such studies should be encouraged because it targeted more precisely the brain region known to be affected in ADHD and could increase the efficacy of EEG-NF (Micoulaud-Franchi et al., 2014).

Lastly, the non-inclusion of Arnold et al. (2013) study in our meta-analysis could be criticized. Nonetheless, as the authors highlighted in their limitation (Arnold et al., 2013), the protocol used was not based on the basic learning theory (in particular by the type of reinforcement) used in standard EEG-NF protocol (Sherlin et al., 2011).

This debate, concerning the choice of studies included in EEG-NF meta-analysis, highlights the importance of investigating the efficacy of EEG-NF in children with ADHD with adequately blinded studies as well as EEG-NF protocols that carefully control the implementation and embedding of training concerning the EEG target, reward feedback, learning during the sessions and transfer learning in daily life (Sherlin et al., 2011; Vollebregt et al., 2014b).

Another possible limit of our approach was to link probably blinded assessments to teacher assessments and probably unblinded assessments to parent assessments. Bralten et al. (2013) observed that the associations with genetics were stronger for parent assessment of ADHD symptoms than for teacher assessments. Because of the few number of studies using EMG-biofeedback or sham-NF as control group, we lacked the possibility to provide the meta-analysis with probably blinded parent assessments. Such studies are to be strongly encouraged and could afford more reliable and valid assessments than probably blinded teacher assessments to evaluate the efficacy of EEG-NF (Bralten et al., 2013).

In conclusion, this meta-analysis using stringent inclusion criteria is the third EEG-NF intervention that confirms the efficacy of EEG-NF when ADHD symptoms are assessed by parents (e.g., with a unblinded assessment). This is also the first meta-analysis that suggests the persistence of EEG-NF efficacy only for the inattention dimension of ADHD when considering recent well-controlled studies that include semi-active and sham-NF controls, as well as probably blinded assessment of inattention symptoms.

## Conflict of interest statement

The authors declare that the research was conducted in the absence of any commercial or financial relationships that could be construed as a potential conflict of interest.
